# Tomato (*Solanum lycopersicum*) *SlIPT4*, encoding an isopentenyltransferase, is involved in leaf senescence and lycopene biosynthesis during fruit ripening

**DOI:** 10.1186/s12870-018-1327-0

**Published:** 2018-06-05

**Authors:** Yong Zhang, Zhengguo Li, Yun Tu, Wenjing Cheng, Yingwu Yang

**Affiliations:** 1 Bioengineering College, Chongqing University, Chongqing, 400044 China; 2 School of Life Sciences, Chongqing University, Chongqing, 400044 China

**Keywords:** Carotenoids, Cytokinins, Fruit ripening, Leaf senescence, Lycopene, *SlIPT4*, Tomato

## Abstract

**Background:**

Lycopene is an important carotenoid pigment in red fruits and vegetables, especially in tomato. Although lycopene biosynthesis and catabolism have been found to be regulated by multiple factors including phytohormones, little is known about their regulatory mechanism. Cytokinins are crucial to various aspects of plant growth. Isopentenyltransferases (IPTs) catalyze the initial rate-limiting step of cytokinins biosynthesis, however, their roles in fruit ripening remain unclear.

**Results:**

Here, the functions of *SlIPT4*, encoding an isopentenyltransferase, were characterized via RNAi-mediated gene silencing in tomato. As we expected, silencing of *SlIPT4* expression resulted in accelerated leaf senescence. However, down-expression of *SlIPT4* generated never-red orange fruits, corresponding with a dramatic reduction of lycopene. Among lycopene biosynthesis-related genes, the fact of remarkable decrease of *ZISO* transcript and upregulation of other genes, revealed that *SlIPT4* regulates positively lycopene biosynthesis via directly affecting *ZISO* expression, and also supported the existence of regulatory loops in lycopene biosynthesis pathway. Meanwhile, the accumulation of abscisic acid (ABA) was reduced and the transcripts *PSY1* were increased in *SlIPT4-RNAi fruits*, supporting the feedback regulation between ABA and lycopene biosynthesis.

**Conclusion:**

The study revealed the crucial roles of *SlIPT4* in leaf senescence and the regulatory network of lycopene biosynthesis in tomato, providing a new light on the lycopene biosynthesis and fruit ripening.

**Electronic supplementary material:**

The online version of this article (10.1186/s12870-018-1327-0) contains supplementary material, which is available to authorized users.

## Background

Carotenoids are a group of terpenoid pigments, naturally synthesized in plants, fungi, algae and photosynthetic bacteria [1], and usually give bright colors to fruit, flower and seed in plants [2, 3]. Carotenoids are more than just pigments, they also act as membrane stabilizers and the precursors of important plant hormones, such as abscisic acid (ABA) and strigolactone, to play important roles in photosynthesis and a variety of physiological processes including plant growth, fruit development and response to abiotic stress [4–6]. Lycopene is a bright red linear carotenoid and widely exists in red fruits and vegetables. Moreover, Lycopene is of particular nutritional interest in promoting health and reducing the risk of various diseases, especially cancer and cardiovascular disease [7–9].

In recent years, significant progress has been achieved in understanding of carotenoid biosynthesis and catabolism using biochemical and genetics approaches [6]. Lycopene is produced from carotenoid biosynthesis pathway, and has been proposed to proceed through a poly-*cis* pathway: geranylgeranyl diphosphate (GGPP) → 15-*cis*-phytoene → 9,15,9′-tri-*cis*-ζ-carotene → 9,9′-di-*cis*-ζ-carotene → prolycopene → all-*trans*-lycopene, catalyzed by phytoene synthase (PSY), phytoene desaturase (PDS), ζ-carotene isomerase (ZISO), ζ-carotene desaturase (ZDS), and carotene isomerase (CrtISO), respectively [2, 3, 10–12]. Lycopene can be further cyclized to produce carotenoids with two β rings (β-carotene) and one β ring and one ε ring (α-carotene). In higher plants, lycopene-β-cyclase (LCYB) and lycopene ε-cyclase (LCYE) catalyze the formation of β-rings and ε-rings, respectively [13, 14]. The α-carotene and β-carotene can be hydroxylated to generate xanthophyll pigments lutein and zeaxanthin, respectively. Zeaxanthin is the precursor of apocarotenoid hormone ABA [3]. Plant carotenoid biosynthesis and catabolism have been found to be regulated by developmental programs, environmental factors, metabolic signals, and classic phytohormones including ethylene, auxin, and ABA [2, 6].

Tomato (*Solanum lycopersicum*) is by far the largest dietary source of lycopene [9], and also a model system for fleshy fruit development and ripening, exhibiting active cell division and expansion at the early developmental stages, and dramatic changes in texture and carotenoid, sugar, and acid content during ripening stages [15]. In tomato fruit, lycopene is the predominant carotenoid pigment and principally responsible for the deep-red color, which is the most obvious characteristic of ripe fruit [16]. For lycopene accumulation during tomato fruit ripening, the transcripts of genes modulating lycopene biosynthesis are upregulated, on the contrary, the expression of genes encoding the enzymes that metabolize lycopene are dramatically decreased [17]. Two tomato mutants altering lycopene content have been characterized: *yellow-flesh* (*locus r*) exhibits yellow fruits as a result of a loss-of-function mutant of the *PSY1* gene [18], and *tangerine* mutant (*locus t*) appears orange fruits due to a mutation in the *CrtISO* gene [19]. Additionally, several transcription factors and genes are involved in the regulatory network of carotenoid biosynthesis. The ripening inhibitor (RIN), a MADS box transcription factor, is able to affect the accumulation of lycopene by directly interacting with the promoter of *PSY1* gene [20]. A STAY-GREEN protein SGR1 can regulate lycopene accumulation through directly interacting with PSY1 to inhibit its activity during tomato fruit maturation [21]. RNAi-mediated silencing of *AP2a*, encoding a tomato APETALA2/Ethylene Responsive Factor (AP2/ERF) transcription factor, reduced total carotenoids accumulation and downregulated the expression of *PSY1* [22]. In *Arabidopsis*, the *AtAP2* and phytochrome-interacting factor 1 (AtPIF1) regulate carotenoid biosynthesis by directly binding to the promoter of *AtPSY1* gene [23, 24]. Although these genes were reported to regulate carotenoid biosynthesis, they focused mainly on repressing the *PSY1* expression in plants. The knowledge on the exact regulation mechanism of carotenoid/lycopene biosynthesis in response to various developmental and environmental factors and so on is still limit so far.

The phytohormone cytokinins (CKs) play crucial roles in a wide aspects of plant growth and development, including cell division, fruit development, leaf senescence, apical dominance, lateral root formation, and stress tolerance [25–28]. Throughout fruit ontogeny, it is known that CKs and their biosynthesis-related genes may play important roles in fruit set and development, however, to our knowledge, little is known about their impact on fruit ripening. The adenosine phosphate-isopentenyltransferases (IPTs) catalyze the initial step of CK biosynthesis to produce isopentenyladenine (ip) nucleotides as CK precursors, it is the rate-limiting biosynthetic step of CKs [29, 30]. Plant *IPTs* belong to multigenic family and have been identified in *Arabidopsis* (*AtIPT1*-*AtIPT9*) [30] and tomato (*SlIPT1*-*SlIPT6*) [27]. Up to now, about the physiological significance of the six tomato *SlIPTs*, only *SlIPT3* and *SlIPT4* were reported to mediate salt stress response in tomato [31]. However, the authors only obtained the *SlIPT3* overexpression tomato lines, and didn’t generate any transgenic tomato plants of *SlIPT4* overexpression or knockdown. So the physiological function of tomato *SlIPT4* is still unknown.

In the present study, we report that the tomato *ISOPENTENYLTRANSFERASE4* gene (*SlIPT4,* GenBank accession number AB690814), is involved in leaf senescence and pigment formation of ripe fruit. RNAi-mediated silencing of *SlIPT4* accelerated leaf senescence and generated orange fruits with a significant reduction of lycopene content. The dramatic reduction of *ZISO* transcripts, moderate decrease of *ZDS* mRNAs, and upregulation of other genes (*PSY1*, *PDS*, *CrtISOs*) in *SlIPT4*-silenced fruits, suggest that *SlIPT4* controls lycopene biosynthesis via directly affecting *ZISO* expression, and also support the existence of regulatory loops in the carotenoids biosynthesis pathway. These data uncover that *SlIPT4* is involved in the regulatory network of lycopene biosynthesis and plays a crucial role in color formation during fruit ripening in tomato.

## Methods

### Plant materials and growth condition

Tomato (*Solanum lycopersicum* cv. MicroTom) plants were grown under the following conditions: 14/10 h day/night cycle, 25 °C/20 °C day/night temperature, 80% humidity, and 250 μmol m^− 2^ s^− 1^ light intensity. For analyzing the organ-specific expression profiling of *SlIPT4*, the roots, stems, leaves, flowers, and fruits (mixture of mature green stage) were collected from 8-week-old wild-type tomato plants. Samples taken from the different parts (ovary, stamen, petal, and sepal) of flowers were harvested at bud (− 2 dpa, day post anthesis), anthesis (0 dpa), and post-anthesis (4 dpa) stages, respectively. The developmental stages of fruits investigated in this study were early mature green (25 dpa), mature green (35 dpa), break (Br, the first visible sign of carotenoid accumulation is evident in surface, 40 dpa), orange (Br + 3 days), and ripening (Br + 7 days).

### Quantitative real-time PCR

Total RNA samples were extracted using Trizol reagent (Invitrogen, USA) according to the manufacturer’s instructions. The first-strand cDNA synthesis was performed using 1 μg of total RNA by PrimeScript™ RT reagent Kit with gDNA Eraser (Takara, Japan). Quantitative real-time PCR (qRT-PCR) was performed using cDNAs corresponding to 2 ng of total RNA in 10 μL reaction volume using SYBR GREEN PCR Master Mix on a CFX96 Touch™ Real-Time PCR Detection System (Bio-Rad, USA). The qRT-PCR reactions were performed as follow: 95 °C for 2 min, followed by 40 cycle of 95 °C for 15 s and 58 °C for 40 s and one cycle of 95 °C for 15 s and 60 °C for 15 s. The 2^-ΔΔCt^ method was used for the analysis of relative gene expression levels as described by Yang et al. [32]. For all qRT-PCR experiments, at least three biological replicates were performed and each reaction was run in triplicate. Tomato *Slactin-51* (GenBank accession number Q96483) was used as the reference gene [32]. The sequences of gene primers for qRT-PCR were listed in Additional file 1: Table S1.

### RNA interference (RNAi) vector construction and plant transformation

A 340 bp specific fragment of *SlIPT4* was amplified from tomato cDNA using the following primers: F 5′-GG*GGTACCAAGCTT*TGCTGAATTGTCAAATTCCGTGG-3′ with *Kpn* I and *Hind* III restriction sites, and R 5′-CCG*CTCGAGTCTAGA*ATAGTGAGATGCTGCTGCCA-3′ with *Xho* I and *Xba* I restriction sites. The PCR products were cloned into the pHANNIBAL vector in the sense orientation and anti-sense orientation into the *Hind* III-*Xba* I polylinker and the *Kpn* I-*Xho* I polylinker, respectively. Then the intron-spliced hairpin construct of *SlIPT4* specific fragment under the transcriptional control of constitutive CaMV35S promoter and OCS terminator was subcloned as *Spe* I*-Sac* I fragment into pCAMBIA1301 binary vector, in which the *hygromycin resistance* gene has been replaced by the *neomycin phosphotransferase II* (*nptII*) gene. Transgenic plants were generated by *Agrobacterium tumefaciens*-mediated transformation. The positive transgenic lines were checked by histochemical β-glucuronidase (GUS) straining and PCR, and the silencing efficiency of *SlIPT4* gene were detected by qRT-PCR.

### Drought treatment

The 4-week-old wild-type tomato plants were stopped watering. When the leaves showed serious wilting, the leaves of drought stress plants and control plants supplied water normally were collected, respectively. Subsequently, the drought treatment plants were supplied water again, and the leaves of 2, 6, 12 and 24 h after watering were collected during the recovery process. The treatments were performed with three biological replicates.

### Chlorophyll and carotenoids measurements

Chlorophyll and carotenoids were measured as described by Forth and Pyke [33]. Briefly, total chlorophyll from 2 g of fresh expanded leaves and total carotenoids from 3 g of fresh ripe fruits were extracted using hexane/acetone (3:2, *v*/v) and acetone/petroleum ether (1:4, v/v), respectively. After centrifugation, the supernatant was measured using spectrophotometer (PerkinElmer, USA). The amount was calculated with the following equations: total chlorophyll mg mL^− 1^ = 8.02 (OD_643_) + 20.2 (OD_647_), and total carotenoids mg mL^− 1^ = (OD_450_)/0.25.

For quantification of lycopene, β-carotene and lutein, pigments were extracted from 2 g of fresh ripe fruits using acetone/petroleum ether (1:1, *v*/v), then dried under a stream of N_2_ and dissolved in 100% dichloromethane. The HPLC analysis was performed with 10 μL dichloromethane-dissolved pigments on ACQUITY UPLC (Waters, USA). Lycopene, β-carotene and lutein were identified by their characteristic absorption spectra (472 nm, 450 nm and 446 nm, respectively) and distinctive retention times, compared with their corresponding standard substance (Sigma, USA). Each carotenoid content was calculated through the linear regression equation generated from the corresponding calibration curve, which was made using the corresponding standard substance. Individual tissue samples above were taken from 3 to 5 leaves or fruits for each line in triplicate.

### ABA measurement

ABA was extracted from 100 mg of pericarp tissues of fresh ripe fruits (Br + 7 days) using 1 mL of solution I (80% methanol, 19.95% H_2_O and 0.05% acetic acid, *v*/v). The supernatant was collected, dried under a stream of N_2_ and dissolved in 0.5 mL petroleum ether to remove the pigments. Then the subnatant was collected, dried under a stream of N_2_ and dissolved in 0.5 mL solution II (40% methanol, 59.94% H_2_O and 0.06% acetic acid, v/v). The HPLC analysis was carried out with 10 μL solution II-dissolved ABA using ultraviolet/visible detector on ACQUITY UPLC (Waters, USA). Spectra were collected at 254 nm, and ABA contents were calculated through the linear regression equation generated from the calibration curve, which was made using the standard substance of ABA (Sigma, USA). Individual tissue samples were taken from 3 to 5 fruits for each line in triplicate.

## Results

### *SlIPT4* expression is predominant in ovary, sustained enhancement during fruit ripening, and regulated by drought stress

**Fig. 1:**
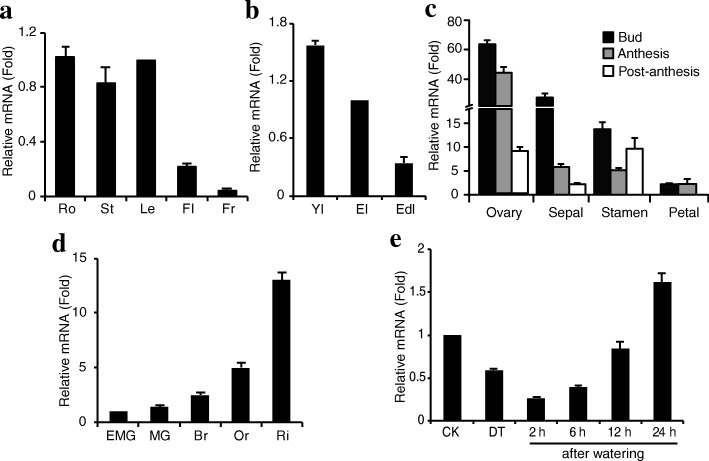
Expression patterns of *SlIPT4* in tomato. Relative expression analysis of *SlIPT4* was performed in different tissues (**a**), in leaves at different developmental stages (**b**), in different parts of flower at different developmental stages (**c**), in fruits at different stages (**d**), and in response to drought stress treatment (**e**) by qRT-PCR. Petals have been shed at the post-anthesis stage, so no data was shown at this stage. Data are expressed as relative values, based on the values of leaf (**a**), expending leaf (**b**), early mature green fruit (**c** and **d**), and corresponding control groups (**e**) taken as reference sample set to 1. Each value represents mean ± SE (standard error) of three replicates. Ro, root; St, stem; Le, leaf; Fl, flower; Fr, fruit. Yl, young leaf; El, expending leaf; Edl, expended leaf. EMG, early mature green; MG, mature green; Br, break; Or, orange; Ri, ripening. CK, control; DT, drought treatment; 2, 6, 12 and 24 h represent the hours after watering

Knowing the expression patterns of a gene sometimes is helpful for knowledge about its physiological function, thus the levels of *SlIPT4* expression in tomato were comprehensively examined using qRT-PCR. In about 8-week-old tomato plants, *SlIPT4* mRNA was detectable in all organs, and shown strong abundance in roots, stems and leaves, moderate accumulation in flowers and weak level in fruits (Fig. 1a). Given the confirmed roles of CKs in the process of leaf senescence and at the early stages of fruit development, *SlIPT4* transcripts were examined throughout leaf and fruit ontogeny. The mRNA accumulation of *SlIPT4* is high in young leaves, and distinctly and continuously downregulated along with the process of leaf development and maturation (Fig. 1b). In flower, *SlIPT4* expression is predominant in ovary, where *SlIPT4* mRNA keeps enormous accumulation at bud (− 2 dpa) and anthesis (0 dpa) stages, and displays dramatic downregulation from anthesis to post-anthesis (4 dpa) transition when fruit set is expected to occur (Fig. 1c). Subsequently, *SlIPT4* expression maintains a moderate level at the mature green stage of fruit development. Interestingly, *SlIPT4* expression displays a sustained enhancement along with fruit ripening, especially, obvious sharp upregulation from orange stage to red stage when lycopene biosynthesis is high active (Fig. 1d), which provides an important clue about its potential roles in the process of tomato fruit ripening.

The expression of *SlIPT4* was significantly decreased by 40% under drought stress compared to the control plants (Fig. 1e). Interestingly, *SlIPT4* mRNA didn’t return toward the normal level at once after recovering watering, on the contrary, it decreased more seriously. After 6 h of restoration, the *SlIPT4* transcripts increased gradually and reached 1.6 fold of that in the control plants at 24 h. The results suggest that *SlIPT4* may be involved in plant response to drought stress, where the phytohormone ABA is the best known trigger of drought tolerance in plants [34].

### *SlIPT4* knockdown in tomato accelerated leaf senescence

**Fig. 2:**
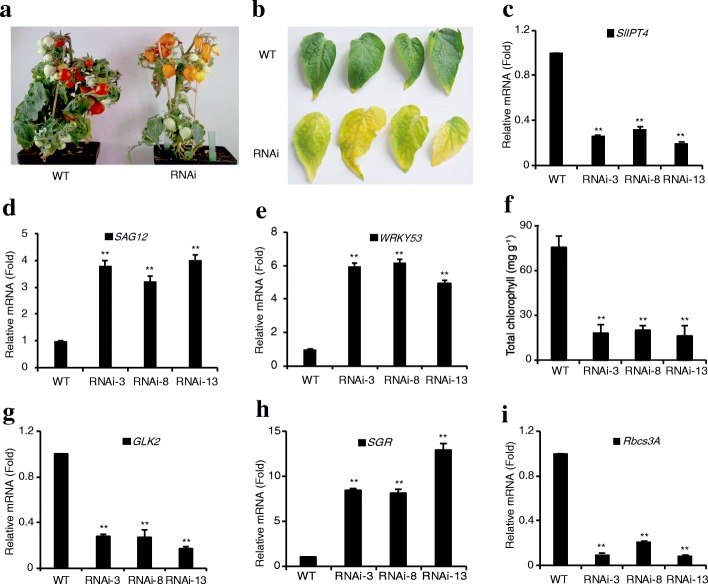
*SlIPT4* knockdown in tomato induced leaf senescence. **a** and **b** The phenotype of accelerated leaf senescence in *SlIPT4*-RNAi lines. Most leaves withered and abscised at 8-week-old plants (**a**), and leaves at expanded mature stage became yellow color (**b**). **c** *SlIPT4* silencing efficiency was confirmed by qRT-PCR. **d** and **e** Relative expression levels of senescence marker genes, *SAG12* and *WRKY53*, were tested by qRT-PCR*.* **f** Obvious reduction of total chlorophyll in expanded leaves of *SlIPT4*-RNAi lines as shown in **b**. Biological replicates were performed in triplicate, and the data are shown as mean ± SE. **g** and **h** and **i** Relative expression levels of *GLK2*, *SGR*, and *Rbcs3A* (regulating chlorophyll biosynthesis, chlorophyll degradation, and photosynthetic rate, respectively) were tested by qRT-PCR. For qRT-PCR, data are expressed as relative values based on the values of the control wild-type (WT) taken as reference sample set to 1. Each value represents mean ± SE of three replicates. Asterisks represent significant differences between *SlIPT4*-RNAi line and wild-type control (*P < 0.05, **P < 0.01, Student’s t test)

To characterize the physiological function of *SlIPT4* in tomato, a loss-of-function approach was implemented using RNAi strategy. A total of more than ten transgenic lines were generated via *Agrobacterium tumefaciens*-mediated transformation. The most readily visible phenotype was related to leaf senescence. The wild-type tomato leaves were still green and alive at later growth stage of about 8-week-old plants (Fig. 2a). By contrast, the leaves of *SlIPT4*-RNAi transgenic lines turned into yellow color at expanded mature stage and displayed an accelerated senescence phenotype (Fig. 2b), and most of leaves withered and abscised from plants at 8-week-old stage (Fig. 2a). The level of *SlIPT4* transcript was significantly reduced by more than 70% in transgenic lines compared with that in wild-type control plants (Fig. 2c), further supporting that downregulation of *SlIPT4* accounted for the phenotypes displayed in the transgenic lines. The senescence specificity of senescence-associated gene *SAG12*, encoding a cysteine protease, makes this gene as a molecular marker to study the senescence process [35]. The transcription factor WRKY53 regulates senescence specific gene expression [36]. Further the remarkable enhanced expression of *SAG12* (GenBank no. XM_004233006) and *WRKY53* (GenBank no. XM_004244582), indicated the accelerated senescence occurred in *SlIPT4*-RNAi leaves (Fig. 2d and e).

Knowing chlorophyll is predominant pigments in green leaves, plays an essential role in photosynthesis, and is degraded during the process of leaf senescence, it prompted us to measure chlorophyll content in leaves. At the expanded mature stage as shown in Fig. 2b, *SlIPT4* silencing resulted in an about 75% reduction of total chlorophyll content in yellow leaves of *SlIPT4*-RNAi lines compared with that in green leaves of wild-type control plants (Fig. 2f). Considering chlorophyll homeostasis is mainly maintained through the dynamic balance between biosynthesis and degradation in plants, the expression levels of two crucial genes regulating these processes were determined by qRT-PCR. The transcription factor Golden2-like (GLK2) regulates positively chlorophyll biosynthesis through affecting chloroplast development [37]. STAY-GREEN (SGR) plays a decisive impact on chlorophyll degradation in plants [38]. Agreeing with the accelerated-senescence phenotype and the reduced chlorophyll accumulation in *SlIPT4*-RNAi leaves, the transcripts of *GLK2* were remarkably decreased (Fig. 2g), while *SGR* expression was significantly upregulated (Fig. 2h), suggesting that *SlIPT4* silencing could disturb chlorophyll accumulation through blocking chlorophyll biosynthesis and promoting the degradation of chlorophyll in leaves. The decrease of photosynthetic rate is also an important characteristic of leaf senescence. The dramatic downregulated expression of a key gene, *ribulose-1,5-bisphosphate carboxylase small subunit* (*Rbcs3A*) (Fig. 2i), in regulating photosynthetic rate [39], indicated a reduced photosynthetic rate in *SlIPT4*-RNAi leaves. It is well known that CKs can inhibit leaf senescence [40], however, its exact regulation mechanism is still unknown. Here phenotype and molecular analyses of *SlIPT4*-RNAi transgenic tomatoes support the hypothesis that *SlIPT4* is involved in this regulatory network of leaf senescence.

### *SlIPT4* silencing in tomato caused orange fruits and decreased lycopene accumulation

**Fig. 3:**
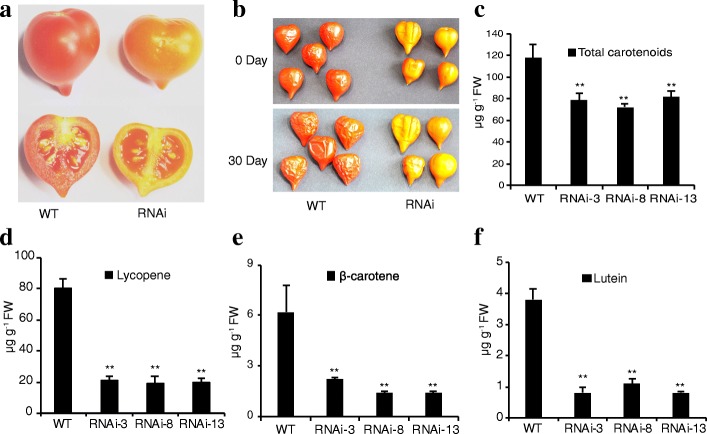
*SlIPT4* downregulation in tomato resulted in orange fruits and decreased lycopene accumulation. **a** and **b** Phenotypes of fruits at about 7 days after break stage (**a**), and after holding on plants until plant died (0 day) and a storage of 30 days at room temperature (**b**). **c**, **d**, **e** and **f** A moderate reduction of total carotenoids (**c**) measured using spectrophotometer, and dramatic decrease of lycopene (**d**), β-carotene (**e**), and lutein (**f**) quantified via HPLC in *SlIPT4*-RNAi orange fruits. FW = fresh weight. The biological replicates (3–5 fruits per sample) were performed in triplicate, and the data are shown as mean ± SE. Asterisks represent significant differences between *SlIPT4*-RNAi line and wild-type control (*P < 0.05, **P < 0.01, Student’s t test)

Although the role of CKs in regulating cell division at the early stages of fruit development is well known, and *SlIPT4* gene exhibits very strong expression level in ovaries and obvious dynamic expression in flowers during bud-to-anthesis and anthesis-to-post-anthesis transitions (Fig. 1c), the behaviors of fruit set and developmental process in all *SlIPT4*-silenced lines were indistinguishable from the control plants. However, surprisingly, the fruits of *SlIPT4*-RNAi lines couldn’t normally ripen and displayed orange surface at about 7 days after breaker stage, while the fruits of wild-type control plants could become typical deep-red at the same growth stage (Fig. 3a). To reveal whether *SlIPT4* downregulation delayed the progress of tomato fruit ripening, the orange fruits were held on the plants. However, the orange fruits of *SlIPT4*-RNAi lines didn’t switch to red color even when plants died (see 0 day fruits in Fig. 3b). Moreover, these orange fruits also couldn’t change the color after a long-time storage of about 30 days at room temperature (Fig. 3b). These results stated clearly that *SlIPT4* is involved in regulating color formation during tomato fruit ripening, and its downregulation caused never red fruits.

Given the dramatic increase of carotenoids, especially lycopene during fruit ripening in tomato, the related carotenoids were measured in both *SlIPT4-*RNAi and wild-type fruits. Compared with wild-type control fruits, the contents of all carotenoids measured were reduced in *SlIPT4*-RNAi fruits (Fig. 3c-f). Total carotenoid levels were reduced by about 35% when quantified using spectrophotometric methods (Fig. 3c). However, HPLC-mediated quantification of lycopene accumulation showed a decrease of more than 75% (Fig. 3d, Additional file 2: Table S2). Due lycopene is the predominant pigment and endows the ripe fruit with deep-red color, we concluded that the enormous reduction of lycopene accounted for the orange color in transgenic fruits. Meanwhile two derivatives of lycopene, β-carotene and lutein, were measured to assess the activity of lycopene catabolism. Similar to lycopene in *SlIPT4*-RNAi fruits, the contents of both β-carotene and lutein were also significantly reduced (Fig. 3e-f, Additional file 2: Table S2), suggesting the low content of lycopene in *SlIPT4*-RNAi fruits was not because of its biological catabolism. Therefore, repression of *SlIPT4* expression leaded to the orange fruit phenotype through inhibiting lycopene biosynthesis.

### Expression of carotenoid biosynthetic genes were altered in *SlIPT4*-silenced fruits

**Fig. 4:**
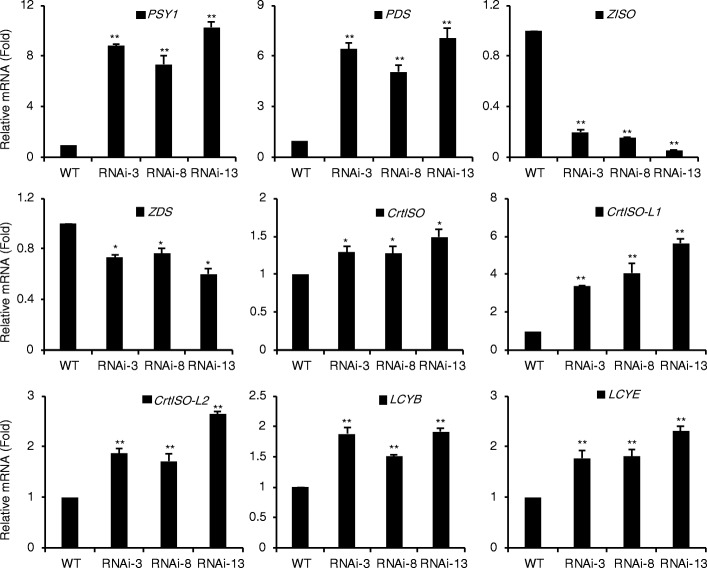
Altered expression of genes in lycopene biosynthesis and metabolism pathway in *SlIPT4*-RNAi orange fruits. The relative level of transcripts were detected via qRT-PCR between wild-type and *SlIPT4* RNAi lines. The data of wild-type (WT) were taken as reference and normalized to 1. Each value represents mean ± SE of three replicates. Asterisks represent significant differences between *SlIPT4*-RNAi line and wild-type control (*P < 0.05, **P < 0.01, Student’s t test)

The carotenoid biosynthesis is mainly regulated at gene transcriptional level during fruit ripening [41]. Accordingly, to detect the mRNA levels of genes involved in lycopene biosynthesis and metabolism pathway is helpful to uncover the molecular regulation mechanism of orange color fruits caused by *SlIPT4* RNAi. As description above, the steps in lycopene biosynthesis pathway are orderly catalyzed by several crucial enzymes encoded by *PSY1*, *PDS*, *ZISO*, *ZDS*, and *CrtISO* genes, respectively (the pathway was shown in Fig. 6). Here the transcript levels of these genes were tested in 5-day fruits after break stage by qRT-PCR (Fig. 4). The first two genes, *PSY1* and *PDS*, being responsible for colorless phytoene synthesis and desaturation to generate 9,15,9′-*cis*-ζ-carotene, were markedly upregulated in *SlIPT4*-RNAi fruits. The *ZISO* gene required for the isomerization of 9,15,9′-*cis*-ζ-carotene to 9,9′-*cis*-ζ-carotene, was significantly downregulated by more than 80% in *SlIPT4*-RNAi fruits. The mRNA of *ZDS*, encoding a desaturase that catalyzes 9,9′-*cis*-ζ-carotene to produce prolycopene, was mildly reduced in *SlIPT4*-RNAi lines. Moreover, *SlIPT4* overexpression under the control of constitutive CaMV35S promoter increased the transcript accumulation of both *ZISO* and *ZDS*, and leaded to a little deep-redder fruits than wild-type control tomatoes (data not shown). The *CrtISO* and two *CrtISO-like* genes (*CrtISO-L1* and *CrtISO-L2*) being responsible for the step of prolycopene to all-*trans*-lycopene in tomato, were upregulated by different degrees in *SlIPT4*-RNAi lines. In conclusion, we thought that the upregulated expression of *PSY1*, *PDS*, and *CrtISO* genes was not the reasons for causing orange fruits in *SlIPT4*-RNAi lines, while the downregulation of *ZISO* and *ZDS*, especially the dramatic decrease of *ZISO* mRNAs, blocked the carotenoid biosynthesis pathway and resulted in the decreased accumulation of lycopene.

In the process of lycopene metabolism, two key cyclases, lycopene β-cyclase (LCYB) and lycopene ε-cyclase (LCYE), catalyze the formation of β-ring and ε-ring, respectively [13, 14]. Although two products of lycopene metabolism, β-carotene and lutein, were reduced as described above (Fig. 3e-f), the expression levels of *LCYB* and *LCYE* were moderately increased in *SlIPT4*-RNAi lines (Fig. 4). This paradox might due the enhanced catabolism of β-carotene and lutein, or that low lycopene accumulation caused the reduction of lycopene metabolism products, which regulated the expression of their related catalyzing enzyme genes through a negative feedback mechanism.

### *SlIPT4* silencing caused a reduction of ABA content and upregulated expression levels of ABA biosynthesis-related genes

**Fig. 5:**
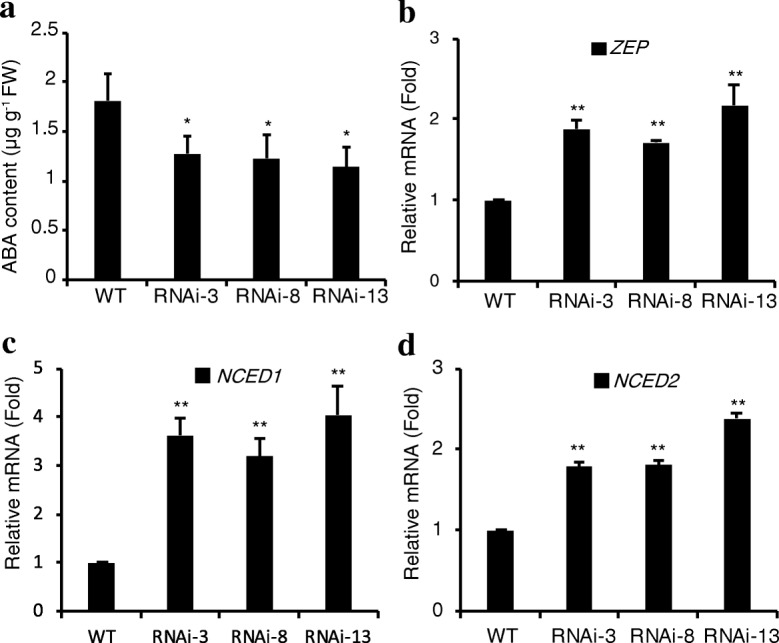
Reduced accumulation of ABA in SlIPT4-RNAi orange fruits. **a** ABA content was measured using HPLC analysis. **b**, **c** and **d** Relative expression levels of ABA biosynthesis-related genes were tested by qRT-PCR. The data of wild-type (WT) were taken as reference and normalized to 1. Each value shown as mean ± SE of three replicates. Asterisks represent significant differences between *SlIPT4*-RNAi line and wild-type control (*P < 0.05, **P < 0.01, Student’s t test)

Considering that the biosynthesis of ABA from the oxidative cleavage of β-carotene is the main pathway in higher plants, and ABA feedback-regulates the carotenoid biosynthesis [3, 42], the ABA content and the expression levels of ABA biosynthesis-related genes were tested to assess whether the decrease of β-carotene content affected ABA biosynthesis in *SlIPT4*-RNAi lines. The obvious reduction of ABA content in *SlIPT4*-RNAi fruits (Fig. 5a), proved that the reason of the low content of lycopene metabolism products is not their catabolism to produce ABA, but their weak biosynthetic activity caused by the very low accumulation of lycopene. In ABA biosynthesis pathway, zeaxanthin epoxidase (ZEP) and 9-*cis*-epoxycarotenoid dioxygenase (NCED) play key roles in higher plants. The ZEP catalyzes the formation of violaxanthin from zeaxanthin [43], and NCED controls the first committed and rate-limiting step of ABA biosynthesis from carotenoid pathway [44]. The upregulation expression of both *NEP* and *NCED* (*NCED1* and *NCED2*) in orange fruits of transgenic lines (Fig. 5b-d), suggested that ABA biosynthesis genes were negative feedback regulated by the level of ABA accumulation.

## Discussion

Leaf senescence is an internally programed phase with the redistribution of micro- and macro-nutrients from leaves to reproductive organs [40, 45–47]. Premature senescence in leaves generally results in reduced crop production and poor grain quality, while delaying leaf senescence can prolong the photosynthesis time and provide sufficient assimilated carbon to grain during the grain-filling period, thereby increasing crop yield. In plants, it has been confirmed that phytohormones are crucial in the regulation of leaf senescence, and among the various plant hormones, CKs have received the maximum attention for their roles in depressing leaf senescence [40]. For instance, exogenous application of CKs has the ability to delay the senescence of detached leaf in dark condition [48]. Although CKs play key roles in regulating leaf senescence, little is known about their exact regulation mechanism. Furthermore, endogenous CKs include several different forms, such as *trans*-zeatin (tZ), isopentenyladenosine (iPR), dihydrozeatin riboside (DZR), and isopentenyladenine (iP), each of them may play different roles in plants. It is puzzled which one or several CKs control the process of leaf senescence. The isopentenyltransferases (IPTs) are the rate-limiting enzymes catalyzing the initial step of CK biosynthesis, and are thought to have a different role in producing different CK among them [49]. Thus it is very necessary to illuminate the functions of *IPTs.* In the present study, we demonstrated that repressing expression of *SlIPT4* accelerated leaf senescence in tomato, which is active and useful to discover the molecular mechanism of CKs regulating leaf senescence.

The prominent visible color change (from green to yellow) of leaf senescence is mainly caused by the degradation of green pigment chlorophyll [50]. Chlorophyll catabolism is a multistep pathway regulated by many factors [40]. And among them, the SGR protein, characterized from *stay green* mutant, plays crucial roles in the regulation of chlorophyll degradation through dismantling photosynthetic chlorophyll-protein complexes and thus allowing chlorophyll-breakdown enzymes to access their substrate [51–55]. Repression of *SGR* expression delayed chlorophyll degradation in tomato fruits and leaves, while constitutive expression of *SGR* accelerated chlorophyll breakdown in leaves [54]. In *SlIPT4*-silenced leaves, the level of *SGR* mRNA was remarkably increased by about 8–12 fold (Fig. 2h), consistent with the decreased content of total chlorophyll (Fig. 2f). Besides enhanced degradation of chlorophyll, the chlorophyll biosynthesis-related gene, *GLK2*, was decreased in *SlIPT4*-silenced leaves (Fig. 2g). Moreover, the decline in photosynthetic capability is also the vital feature of leaf senescence, and the expression of *Rbcs3A*, encoding a positive regulator of photosynthesis, was significantly downregulated (Fig. 3i), suggesting the reduced photosynthesis efficiency in *SlIPT4*-RNAi lines. Taken together, these data indicated that *SlIPT4* is assuredly necessary for controlling leaf senescence.

It was expected that repression of *SlIPT4* expression accelerated leaf senescence. However, it was out of our expectation that *SlIPT4* knockdown did not cause any obvious phenotypic changes during fruit developmental stages, especially the cell division stage. Although the roles of CKs in fruit development are well known, not all types of CKs have effects on this process. For example, after applications of exogenous CKs in unpollinated ovaries of tomato, tZ, 6-benzylaminopurine (BA), and kinetin didn’t have any visible effect on fruit set and growth, it was similar to untreated control ovaries with phenotype of neither abscission nor growth, however, application of N-(2-chloro-pyridin-4yl)-N′-phenylurea (CPPU) induced growth [27]. Thus, we think no visible effect on fruit development in *SlIPT4*-RNAi lines may be due that there is potential functional redundancy among *IPT* genes during fruit development, or this kind of CK produced by *SlIPT4* may not be pivotal for this process.

CKs are thought to be important for fruit development, especially cell division [27], to our knowledge, their roles in fruit ripening have no reports so far. Interestingly, the mRNA level of *SlIPT4* gradually increases during fruit ripening in tomato (Fig. 1d). More surprisingly, repressing the expression of *SlIPT4* resulted in producing orange fruits, which were never red (Fig. 3a-b). As the predominant pigment in ripe tomato fruits, lycopene content was tremendously decreased (Fig. 3d, Additional file 2: Table S2), suggesting *SlIPT4* silencing dramatically effected lycopene biosynthesis. During fruit ripening, chloroplasts convert to chromoplasts, which are pigment-filled plastids responsible for the bright colors. Thus the subcellular localization of SlIPT4 in the chloroplast and the cytosolic area surrounding chloroplasts uncovered by Žižková et al. [31], strongly supports our results that *SlIPT4* is involved in regulating leaf senescence and fruit color formation.

Among lycopene biosynthesis-related genes, *SlIPT4* RNAi resulted in remarkable decrease of *ZISO* mRNA, moderate downregulation of *ZDS* transcript, and obvious upregulated expression of *PSY1*, *PDS*, and *CrtISOs* (Fig. 4). *ZISO* is first defined by the maize y9 and present in single copy in tomato, *Arabidopsis* and grape [10, 56, 57]. In tomato, *ZISO* widely expresses in flower, root, leaf and fruit, and maintains high expression level during fruit ripening [10]. Silencing the expression of *ZISO* via virus-induced gene silencing (VIGS) in tomato resulted in pale-red fruits with a distinct reduction of lycopene and a compensatory increased accumulation of phytoene, phytofluene, and ζ-carotene [10]. Similarly, the recessive mutant *zeta* (*z*^*2803*^), a mutation in *ZISO* gene, blocks carotenoid biosynthesis in fruits at ζ-carotene with almost undetectable lycopene [58]. Moreover, *SlIPT4* overexpression could enhance the mRNA levels of both *ZISO* and *ZDS*, and give rise to a little deep-redder fruits (data not shown). Taken together, these data strongly supported that downregulation of *SlIPT4* expression in tomato fruit inhibited carotenoids biosynthesis, especially caused a sharp reduction of lycopene content, through repressing the expression of *ZISO*.

The puzzling complex regulatory loops regulating the abundance of key transcripts in response to the operation of the carotenoid biosynthesis pathway is existent in tomato, it was discussed previously by Kachanovsky et al. [58] and Fantini et al. [10]. The level of product feedback regulates the transcript induction of its upstream genes in carotenoid biosynthesis pathway. For example, the *PDS* promoter is induced when carotenoid accumulation is repressed in tomato leaves [59]; Prolycopene or its metabolite was hypothesized as the signal mediating *PSY1* induction [58]; Fantini et al. thought a minimum level of prolycopene is required to attain *PSY1* transcript induction [10]. The transcript level of *ZISO* was highly correlated with mRNAs of *PDS*, encoding the enzyme that produces the substrate for *ZISO* [10]. The *ZISO* and *CrtISO* were induced in *PDS*- and *ZDS*-silenced fruits, respectively [10]. The downregulation of *ZISO* and *ZDS*, and the upregulation of other genes (*PSY1*, *PDS*, *CrtISO*, *CrtISO-L1*, and *CrtISO-L2*) involved in carotenoid biosynthesis in *SlIPT4*-silenced fruits with very low content of lycopene (Figs. 3 and 4), supports the hypothesis of the existence of regulatory loops in the carotenoid biosynthesis pathway.

It has been confirmed that ABA is mainly derived from carotenoids pathway in higher plants, and low content of ABA caused by disruption of carotenoid biosynthesis or active biosynthesis of ABA induces *PSY* transcription through feedback regulation mechanism (reviewed in ref. [3, 58]). For example, under abiotic stress of saline or drought, the increased demand for ABA to trigger stress tolerance drives *PSY* expression to enhance carotenoid biosynthesis pathway for providing more xanthophylls of ABA precursors in roots [60, 61]. In *SlIPT4*-RNAi fruits, the ABA accumulation was reduced due to the repression of carotenoid biosynthesis pathway (Fig. 5a), while the upregulated expression of ABA biosynthesis-related genes (*ZEP*, *NCED1* and *NECD2*) (Fig. 5b-d) indicated that the transgenic plants might try to restore the normal level of ABA. Accordingly, the low accumulation of ABA in *SlIPT4*-RNAi fruits was likely to feedback regulate carotenoid biosynthesis pathway for trying to promote the supply of ABA precursors through inducing *PSY1* expression, which accords with this feedback regulation system between ABA content and *PSY1* expression.

The decreased content of CKs in *ipt1 3 5 7* mutants or overexpression transgenic plants of *CKX*, encoding a cytokinin oxidase/dehydrogenase catalyzing the irreversible degradation of CKs, could significantly reduce ABA accumulation in *Arabidopsis thaliana* [62]. *SlIPT4* expression is very sensitive to drought stress (Fig. 1e), where ABA is the best known trigger of drought tolerance in plants [34]. These results suggest that *SlIPT4* may be associated with ABA biosynthesis. Accordingly, both the potential reduction of CK content caused by *SlIPT4* silencing and the sharp repression of carotenoid biosynthesis might result in a reduced accumulation of ABA in *SlIPT4*-RNAi fruits.

**Fig. 6:**
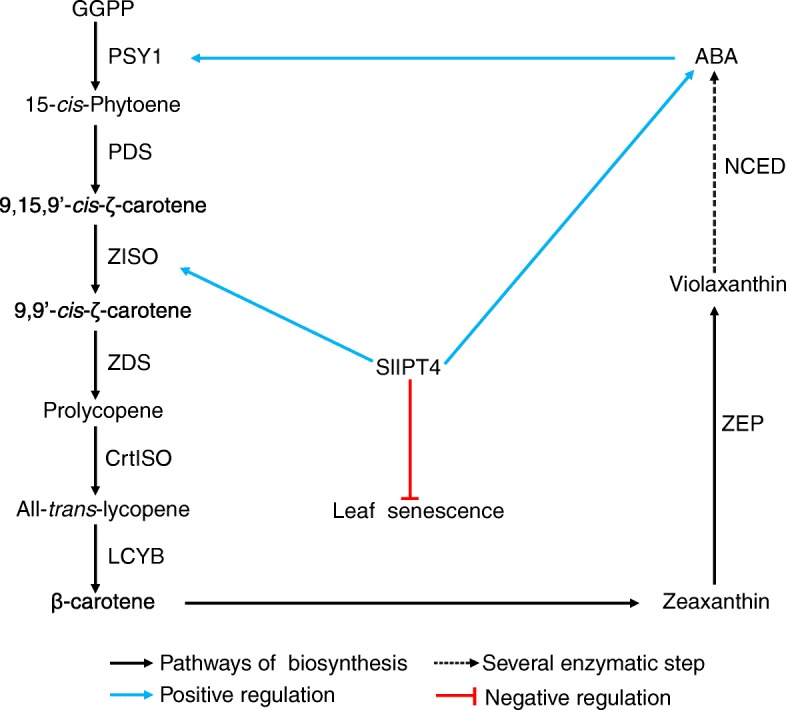
Proposed model for *SlIPT4* regulating lycopene biosynthesis and leaf senescence in tomato. *SlIPT4* regulates the carotenoid biosynthesis via controlling the expression level of *ZISO*, and modulates leaf senescence through affecting the corresponding CK biosynthesis. The alteration of carotenoid biosynthesis affects directly the supply of ABA precursors, and also ABA accumulation is regulated by the correlation between CKs and ABA. ABA feedback regulates the expression level of the first gene *PSY1* in carotenoid biosynthesis pathway

Consequently, based on the results and analysis above, we raised a proposed regulation model for the roles of *SlIPT4* in the control of lycopene biosynthesis and leaf senescence in tomato (Fig. 6). In this model, *SlIPT4* gene regulates the lycopene biosynthesis via controlling the expression level of *ZISO*. The ABA accumulation is regulated by *SlIPT4* through two potential ways: one way is that the alteration of carotenoid biosynthesis affects directly the supply of ABA precursors; and another way is that ABA accumulation is regulated by the potential correlation between CKs and ABA. Then the alteration of ABA content feedback regulates the expression level of the first gene *PSY1* in carotenoid biosynthesis pathway. Though ABA was discovered to be involved in leaf senescence, however, due to the known role of CKs in leaf senescence, we thought that the *SlIPT4*-mediated leaf senescence might be mainly attributed to affect the corresponding CK biosynthesis in tomato. Nevertheless, the exact mechanism through which the effects of *SlIPT4* are exerted in tomato leaves and fruits needs and deserves further investigation.

## Conclusion

Most studies devoted so far to *IPT* genes focused on their roles in the known cytokinin-related process, such as cell division, apical dominance, leaf senescence and fruit development, as well as in response to biotic and abiotic stress*.* Here, we uncovered the functional roles of *SlIPT4* in not only leaf senescence but also the regulation of lycopene biosynthesis in tomato. The downregulation of *SlIPT4* repressed carotenoids biosynthesis and reduced dramatically the accumulation of lycopene in tomato fruits. Among lycopene biosynthesis-related genes, dramatic reduction of *ZISO* transcripts, moderate decrease of *ZDS* mRNAs, and upregulation of other genes (*PSY1*, *PDS*, *CrtISOs*), suggested that *SlIPT4* controls lycopene biosynthesis likely through affecting *ZISO* expression, and also supported the existence of regulatory loops in carotenoids/lycopene biosynthesis pathway. The decrease of ABA content and upregulation of *PSY1* expression in *SlIPT4*-RNAi fruits, implied the feedback regulation between ABA and carotenoids biosynthesis. To our knowledge, this is the first report that cytokinin biosynthesis-related gene is involved in color formation during fruit ripening.

## Additional files

Additional file 1: **Table S1.** Primers used for qRT-PCR. (PDF 37 kb)

Additional file 2: **Table S2.** Carotrnoids in fruits of wild-type and *SlIPT4*-RNAi tomato plants. (PDF 61 kb)

## Abbreviations

ABA: Abscisic acid
CKs: Cytokinins
CrtISO: Carotene isomerase
dpa: Day post anthesis
GLK2: Golden2-like
IPT: Isopentenyltransferases
LCYB: Lycopene-β-cyclase
LCYE: Lycopene ε-cyclase
NCED: 9-cis-epoxycarotenoid dioxygenase
PDS: Phytoene desaturase
PSY: Phytoene synthase
qRT-PCR: quantitative real-time PCR
Rbcs3A: Ribulose-1,5-bisphosphate carboxylase small subunit
RNAi: RNA interference
SGR: STAY-GREEN
ZDS: ζ-carotene desaturase
ZEP: Zeaxanthin epoxidase
ZISO: ζ-carotene isomerase
