# Supplementary material for: Tomato (Solanum lycopersicum) SlIPT4, encoding an isopentenyltransferase, is involved in leaf senescence and lycopene biosynthesis during fruit ripening
Source: BMC Plant Biol. 2018 Jun 5;18:107. doi: 10.1186/s12870-018-1327-0 (PMC5987576; doi:10.1186/s12870-018-1327-0)
Supplement: Supplementary file 1 — Table S1. Primers used for qRT-PCR. (PDF 37 kb) [file 12870_2018_1327_MOESM1_ESM.pdf]

Table S1 Primers used for qRT-PCR

Gene	Forward primer(5'-3')	Reverse primer(5'-3')
SlIPT4	ACTCTCTATCGACTTAGCCACTCAA	ATTACCCCTAGGCAATGATGTGGT
GLK2	AACATCAGCGAGATTTGCAGC	CAACTTTGATGATGGCAGGGC
SGR	CACTTCATGTCCATTGCCAC	ACAAAAGCCTTGAGAACCACA
Rbcs3	TGCTCAGCGAAATTGAGTACCTAT	AACTTCCACATGGTCCAGTATCTG
PSY1	GGGCGGCCATTTGACAT	AATGGCTGAATATCAACTGGAAAGT
ZISO	CCTTCTTCTTCCTATACCCGTCG	AGCGTGTGAGCTAAGCACCA
ZDS	GGTGGGTGCTGAAAAAAAT	GGAAAGCGGAAATCAAGTT
CrtISO	AATGCTGGTAGCATCGCTC	ATTCCGCCAAAATGTCTGTCAC
CrtISO-L1	TCAAGGAGCCCTTGGTGCTA	GGAGATCCAGCCAATTTCGTAT
CrtISO-L2	TTCATCATTCACGCCTCGTC	CATGAACTCGCAGAACTTGTCG
LCYB	CGACGTGATCATTATCGGAGC	GTGGTGAAGGGTCAACACAACA
LCYE	GCCACAGGTTATTCAGTCGTCA	CCAGTCCAAATAGGAAAAACGAT
ZEP	TCTAGGAGGCAATGGGGACA	TCTGCATCAGTAGCACGCTC
NCED1	AGGCAACTGTGAAGCTTCCATCAAG	TCCATTAAAGAGGAATATTCCGGGGAC
NCED2	TGGTTTTCATGGGACATTCATTAGC	ATCTCCCTTCTCAACTCCCTATTCC
WRKY53	TCTCAACCAGCAGCCAGATG	CGTTGTGGAAGTGTTGGCTG
SAG12	CACACCCTAAGATTATGTCCTCCTC	CATCCACATTCCATTTGGTCCTTGA
Slactin-51	TGTCCCTATTTACGAGGGTTATGC	CAGTTAAATCACGACCAGCAAGAT
