# Supplementary material for: Tomato (Solanum lycopersicum) SlIPT4, encoding an isopentenyltransferase, is involved in leaf senescence and lycopene biosynthesis during fruit ripening
Source: BMC Plant Biol. 2018 Jun 5;18:107. doi: 10.1186/s12870-018-1327-0 (PMC5987576; doi:10.1186/s12870-018-1327-0)
Supplement: Supplementary file 2 — Table S2. Carotrnoids in fruits of wild-type and SlIPT4-RNAi tomato plants. (PDF 61 kb) [file 12870_2018_1327_MOESM2_ESM.pdf]

Table S2 Carotrnoids in fruits of wild-type and *SlIPT4*-RNAi tomato plants (μg g^{-1} FW)

Carotenoids	WT	RNAi-3	RNAi-8	RNAi-13
Lycopene	80.3±5.7	20.9±3.0	19.3±4.5	19.7±2.6
β-Carotene	6.2±1.6	2.2±0.15	1.4±0.13	1.4±0.08
Lutein	3.8±0.35	0.8±0.07	1.1±0.05	0.8±0.04
Total	117.5±12.3	78.3±6.5	72.3±2.7	81.5±5.2

Fruit samples are collected at Br+7 days stage. The biological replicates (3-5 fruits per sample) were performed in triplicate, and the data are shown as mean ± SE. FW = fresh weight.
